# Defects in Mitochondrial Clearance Predispose Human Monocytes to Interleukin-1β Hypersecretion

**DOI:** 10.1074/jbc.M113.536920

**Published:** 2013-12-19

**Authors:** Robert van der Burgh, Lotte Nijhuis, Kalliopi Pervolaraki, Ewoud B. Compeer, Lieneke H. Jongeneel, Marielle van Gijn, Paul J. Coffer, Michael P. Murphy, Pier G. Mastroberardino, Joost Frenkel, Marianne Boes

**Affiliations:** From the ‡ Department of Pediatric Immunology and Infectious Diseases, University Medical Center Utrecht, Wilhelmina Children's Hospital, 3584 EA Utrecht, The Netherlands,; the Departments of ¶ Cell Biology and; § Genetics, University Medical Center Utrecht, 3584 EA Utrecht, The Netherlands,; the ‖ Medical Research Council Mitochondrial Biology Unit, Wellcome Trust, Cambridge, CB2 0XY United Kingdom, and; the ** Department of Genetics, Erasmus Medical Center, 3015 GE Rotterdam, The Netherlands

**Keywords:** Autophagy, Interleukin, Mitochondrial DNA, Monocytes, Redox Regulation, Autoinflammatory Disorder, Periodic Fever

## Abstract

Most hereditary periodic fever syndromes are mediated by deregulated IL-1β secretion. The generation of mature IL-1β requires two signals: one that induces synthesis of inflammasome components and substrates and a second that activates inflammasomes. The mechanisms that mediate autoinflammation in mevalonate kinase deficiency, a periodic fever disease characterized by a block in isoprenoid biosynthesis, are poorly understood. In studying the effects of isoprenoid shortage on IL-1 β generation, we identified a new inflammasome activation signal that originates from defects in autophagy. We find that hypersecretion of IL-1β and IL-18 requires reactive oxygen species and is associated with an oxidized redox status of monocytes but not lymphocytes. IL-1β hypersecretion by monocytes involves decreased mitochondrial stability, release of mitochondrial content into the cytosol and attenuated autophagosomal degradation. Defective autophagy, as established by ATG7 knockdown, results in prolonged cytosolic retention of damaged mitochondria and increased IL-1β secretion. Finally, activation of autophagy in healthy but not mevalonate kinase deficiency patient cells reduces IL-1β secretion. Together, these results indicate that defective autophagy can prime monocytes for mitochondria-mediated NLRP3 inflammasome activation, thereby contributing to hypersecretion of IL-1β in mevalonate kinase deficiency.

## Teaser

**Background:** Periodic fever syndromes are caused by deregulation of interleukin-1β release.

**Results:** Defective autophagy leads to accumulation of damaged mitochondria in monocytes.

**Conclusion:** Mitochondrial components in the cytosol cause priming of monocytes for interleukin-1β release.

**Significance:** The molecular mechanism behind deregulated cytokine secretion provides new clues for intervention.

## Introduction

Periodic fever syndromes are characterized by inflammation that occurs in the absence of apparent infection or high titer autoantibodies (1, 2). In most periodic fever syndromes, the generalized inflammation is driven by IL-1β generated through proteolytic cleavage by the NLRP3 inflammasome. An additional feature associated with inflammation is the production of reactive oxygen species (ROS)^3^ (3, 4). During infection, ROS produced by NADPH-oxidase subunits at the plasma membrane are beneficial because ROS in phagosomes contribute to the killing of intracellular pathogens (5). In several inflammatory diseases, including periodic fever syndromes, ROS levels are increased in the cytosol and contribute to pathology (6). ROS play roles as intracellular second messengers and have immediate effects on the intracellular redox status. In TNF receptor 1-associated periodic syndrome (TRAPS), for example, mitochondria-derived ROS facilitate inflammatory cytokine production (7). Mitochondria are believed to be the main source of ROS in several autoinflammatory disorders (8).

ROS are normally generated within mitochondria as byproducts of oxidative phosphorylation, but when liberated in the cytosol ROS can facilitate activation of the NLRP3 inflammasome (9–11). Normally, mitochondrial contents, including ROS and mitochondrial DNA (mtDNA) are prevented from reaching the cytosol because damaged mitochondria are swiftly neutralized by autophagy (12). During autophagy cytosolic constituents are enclosed within a double-layered lipid membrane vesicle geared to fuse with lysosomes for degradation and recycling of the internal contents (12). Impaired autophagy may interfere with mitochondrial turnover and stimulate NLRP3 activation and IL-1β release, as was shown in murine sepsis models (11), indicating the possibility of a link between defective mitochondrial clearance and autoinflammatory disease. Furthermore, in mice, mitochondria-derived ROS contributes to the activation of the NLRP3-inflammasome-mediated activation of caspase-1 by LPS and ATP (11).

We studied the potential contribution of disordered mitochondrial biology in to IL-1β mediated inflammation in the human monogenetic periodic fever disorder, mevalonate kinase deficiency (MKD), which gives rise to the hyper-IgD and periodic fever syndrome. MKD is caused by loss of function of mevalonate kinase, an enzyme in the mevalonate pathway involved in cholesterol and non-sterol isoprenoid synthesis. The defect seriously impairs isoprenoid biosynthesis, resulting in reduced prenylation of proteins, particularly of some small GTPases. Individuals suffering from MKD experience recurring fever episodes that are to a large extent mediated by IL-1β (13). In this study, we aimed to clarify underlying mechanisms that cause hypersecretion of IL-1β. We show that impaired isoprenoid biosynthesis interferes with autophagy, attenuates the activity of the mitochondrial respiratory chain, and increases the release of mtDNA into the cytosol, thereby fostering an oxidized cytosolic milieu, which ultimately leads to hypersecretion of IL-1β.

## MATERIALS AND METHODS

### 

#### 

##### Reagents

Simvastatin, geranylgeranylpyrophosphate, ATP, *N*-acetyl cysteine, diphenylene iodonium, apocynin, and bafilomycin A_1_ were purchased from Sigma-Aldrich. MitoTracker Green, MitoTracker Deep Red, MitoSOX, tetramethyl rhodamine methyl ester (TMRM), and H_2_DCFDA were purchased from Invitrogen. A Cyto-ID autophagy detection kit was purchased from Enzo Life Sciences. LPS (*Escherichia coli* EH 100) was obtained from Alexis Biochemical. MitoQ and decyltriphenylphosphonium (TPP) were synthesized as described (7). Simvastatin was hydrolyzed to its bioactive form as described previously (14). ATP solution was made in RPMI 1640 and was buffered to a pH of 7.5.

##### Patient Samples

Patients were children between the ages of 2 and 16 years with hyper-IgD periodic fever syndrome caused by compound heterozygous mutations affecting both alleles of mevalonate kinase. All had residual mevalonate kinase activities between 0.1 and 8.5% of healthy controls. At scheduled outpatient visits, patients who were afebrile and well underwent routine blood analysis. The ethical committee of the University Medical Center Utrecht approved the use of residual material for this study. After informed consent was obtained from parents and from patients 12 years and older, residual material from routine blood tests was used to obtain peripheral blood mononuclear cells (PBMCs). PBMCs from patients and healthy donors were isolated using Ficoll density gradient. PBMC fraction was washed twice in RPMI supplemented with 2% FBS and used immediately.

##### Cell Cultures

THP-1 and HEK293T cells were both cultured in RPMI 1640 supplemented with 1% glutamine, antibiotics (penicillin, streptomycin), and 10% FBS. Simvastatin treatment of cells was 48 h prior to the start of the experiment and at a concentration of 10 μm unless stated otherwise in the figure legends.

##### Mitochondrial Damage, Potential, and Superoxide Measurements

Cells were washed once in PBS and resuspended in RPMI (without phenol red and without FBS) and appropriate probe. Staining concentrations were 50 nm MitoTracker Green, 50 nm MitoTracker Deep Red, 20 nm TMRM, and 5 μm MitoSOX. Cells were incubated in the dark for 30 min at 37 °C. Cells were centrifuged (500 × *g* for 5 min) and suspended in RPMI without phenol red with 10% FBS. Cells were kept in the dark until measurement on FACS CANTO-II. Analysis was done with FACS Diva software.

##### Oxygen Consumption and Glycolysis Measurements

Oxygen consumption rate and glycolysis were measured using the Seahorse XF^e^24 extracellular flux analyzer (Seahorse Biosciences) according to the manufacturer's instructions. THP-1 cells were bound to the well using BD Cell-TAK coating. Coating of the wells was done according to the manufacturer's instructions.

##### RNA Isolation and Quantification

RNA was isolated by dissolving cell pellets in TRIpure (RnD) and following manufacturer's protocols. Isolated RNA was converted to cDNA using iScript (Bio-Rad) according to manufacturer's instructions. Detection was done with CF-96 (Bio-Rad) using SYBR green (Bio-Rad), 100 ng of cDNA was used per reaction. The primers used were heme oxygenase-1 (HO-1) forward, 5′-TCAGGCAGAGGGTGATAGAAG-3′; HO-1 reverse, 5′-TTGGTGTCATGGGTCAGC-3′; ATG7 forward, 5′-CAGTTTGCCCCTTTTAGTAGTGC-3′; ATG7 reverse, 5′-CCTTAATGTCCTTGGGAGCTTCA-3′; B_2_M forward, 5′-CCAGCAGAGAATGGAAAGTC-3′; B_2_M reverse, 5′-GATGCTGCTTACATGTCTCG-3′; GAPDH forward, 5′-GTCGGAGTCAACGGATT-3′; and GAPDH reverse, 5′-AAGCTTCCCGTTCTCAG-3′.

##### ATG7 shRNA Knockdown

Two different short hairpin RNA sequences (Sigma-Aldrich) in a lentiviral vector (MISSION pLKO.1-puro) were used to make lentiviral particles. THP-1 cells were infected twice and then selected for puromycin resistance. ATG7 knockdown (KD) was tested with quantitative PCR for ATG7. Hairpin sequences were ATG7 KD1, 5′-CCGGGCCTGCTGAGGAGCTCTCCATCTCGAGATGGAGAGCTCCTCAGCAGGCTTTTT-3′; ATG7 KD2, 5′-CCGGGCTTTGGGATTTGACACATTTCTCGAGAAATGTGTCAAATCCCAAAGCTTTTT-3′; and control (scrambled), 5′-CCGGCAACAAGATGAAGAGCACCAACTCGAGTTGGTGCTCTTCATCTTGTTGTTTTT-3′. KD efficiency was determined by quantitative PCR.

##### Cytosolic Mitochondrial DNA Measurement

Protocol was adapted slightly from Ref. 15. Cultured cells (1.5 × 10^7^) were washed twice in ice-cold PBS and then homogenized with a Dounce homogenizer in ice-cold 100 mm Tris-HCl (pH 7.4) containing 0.25 m sucrose, 1 mm EDTA, and protease inhibitor mix. Samples were centrifuged (700 × *g* at 4 °C) for 10 min, and the supernatant was collected and kept on ice. Protein content was determined by BCA protein assay. Samples were normalized to volume and protein concentration and centrifuged (10,000 × *g* at 4 °C) for 30 min. Supernatant was collected, and 200 μl was used to isolate DNA with DNA blood and tissue kit (Qiagen). Mitochondrial DNA copy number was determined by quantitative PCR for cytochrome *b*. The following primers were used: CYTB forward, 5′-GCCTATATTACGGATCATTTCTCTACT-3′; and CYTB reverse, 5′-GCCTATGAAGGCTGTTGCTATAGT-3′.

##### Glutathione Measurement

Glutathione and glutathione disulfide were colorimetrically measured as described previously (9). For glutathione disulfide, 2.5 times more sample was used to achieve accurate readings. Ratio measurements were corrected accordingly.

##### Antioxidant Capacity Measurement

Cells were centrifuged and resuspended in staining mix (RPMI without phenol red with 10% FBS, 10 μm H_2_DCFCA) and incubated in the dark for 15 min at 37 °C. Cells were immediately transferred to six FACS tubes, and the fluorescence was measured. After the first fluorescent measurements, hydrogen peroxide was added to the different tubes at a final concentration of 0, 5, 15, 45, 135, and 150 μm, respectively. Tubes were briefly vortexed and incubated for 4 min covered from light and measured two more times in consecutive order. Analysis was performed by calculating the rate of fluorescence increase over time per sample (slope). The ratio of the fluorescence increase (slopes) at the different H_2_O_2_ concentrations over the background slope (without H_2_O_2_) provides a measure of antioxidant capacity (relative ratio). This allows the comparison of measurements by removing technical variation.

##### Cytokine Measurements

Cells were centrifuged (500 × *g* for 5 min) and plated in 96-well plates in triplicates (2.0 × 10^5^ cells/well in 200 μl). Inhibitors were added, followed by 1 h of incubation at 37 °C. Next LPS (200 ng/ml) was added, and supernatants were collected after 4 h and stored at −80 °C until measurement. Cytokine concentrations were determined by Mulitplex bead analysis.

##### Immunoblot Analysis

Cells were washed twice in PBS and then resuspended in Laemmli buffer and boiled for 10 min. Samples were then aliquoted and stored at −20 °C until use. Protein content was determined with BCA assay, and samples were diluted to 1 μg/μl. 5% (v/v) β-mercaptoethanol was added to the samples, and they were separated on 12% SDS-PAGE gel, followed by transfer to PVDF-FL membrane. 5% dried nonfat milk was used for blocking followed by primary antibody incubation (overnight 4 °C, 0.5% milk in TBS-T), three washes, and secondary antibody incubation (1 h at room temperature, 0.5% milk in TBS-T). Detection was done with ECL. Antibodies used: anti-p62 (Santa Cruz; sc-28359), anti-LC3 (Nanotools, 0231-100/LC3-5F10), anti-actin (Santa Cruz; sc-1616), and anti-HSP90 (Cell Signaling Technology; 4875S). We performed quantification of signal on blots using ImageJ (National Institutes of Health).

##### Confocal Analysis

HEK 293T cells were seeded in 24-well culture plates on 1.5-mm glass coverslips precoated with poly-l-lysine solution (0.1 (w/v) in H_2_O; Sigma-Aldrich) for 30 min at 37 °C. Plates washed twice in PBS, after which cells were plated at 40% confluency. After 24 h, bafilomycin A1 was added (final concentration, 10 nm; 4 h at 37 °C). The coverslips were washed twice in PBS and fixed (3.7% paraformaldehyde (Merck), 10 min at room temperature). Coverslips were mounted using Mowiol solution containing DAPI. Autophagosomes were counted in a semiautomated manner using Metamorph software (Molecular Devices). LC3-positive regions of interests were derived from binarized images obtained by thresholding immunofluorescence pictures, using the same threshold for all the samples. The number of cells was derived from the number of nuclei.

PBMCs were plated on 8-well Lab-Tek® II Chamber Slides coated with Cell-Tak (BD Biosciences) at a density of 1 × 10^6^ cells/ml. After incubation with rapamycin, the cells were washed twice with PBS and stained with Cyto-ID detection agent according to the manufacturer's instructions. Next cells were washed twice with RPMI 1640 without phenol red, supplemented with 0.2% (v/v) BSA (Roche Applied Science) and 10 mm HEPES, and live cell imaging was performed on a Zeiss LSM710 confocal microscope equipped with a live cell chamber device to maintain 37 °C and 5% CO_2_ condition during experiments.

THP-1 cells were washed with PBS and stained in RPMI (without) phenol red with 100 nm MitoTracker Green and 150 nm MitoTracker Red for 30 min at 37 °C. Cells were washed and plated on WillCo wells coated with Cell-Tak (BD Biosciences) in RPMI (without) phenol red and 10% FBS. All images were obtained with 1.3× optical zoom using Plan-Apochromat 63× 1.40 oil DIC M27 objective on a Zeiss LSM710 and processed using Zen 2009 software (Zeiss Enhanced Navigation).

##### Statistics

The *error bars* shown represent S.E. unless stated otherwise in the figure legends. Statistical test between two variables was done using the Mann-Whitney test. In the figures, one asterisk (*) indicates a *p* value of <0.05, and two asterisks (**) indicate a *p* value of <0.01.

## RESULTS

### 

#### 

##### Altered Redox State in MKD Model Monocytes Exhibiting IL-1β Hypersecretion

Mouse studies support that mitochondrial ROS (mtROS) can mediate the activation of NRLP3 inflammasomes (9, 11, 14). We hypothesized that IL-1β hypersecretion by human monocytic cells in analogy involves increased cytosolic levels of mtROS. To test this hypothesis, we used freshly isolated monocytes from healthy individuals or patients suffering from MKD and THP-1 cells that were rendered similarly isoprenoid-deficient because of 48-h treatment with simvastatin. Simvastatin inhibits the HMG-CoA reductase, the rate-limiting enzyme in the mevalonate pathway, leading to a shortage of isoprenoids. The inclusion of simvastatin in LPS-treated monocytic cultures mimics the IL-1β hypersecretion phenotype seen in the autoinflammatory syndrome MKD (18–22).

**FIGURE 1.**
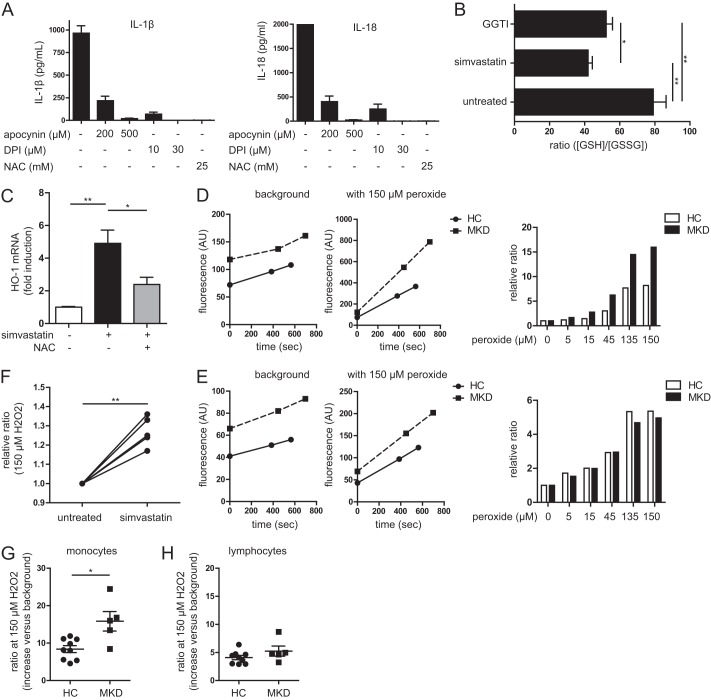
**Altered redox state in periodic fever syndrome model cells and patient monocytes.** *A*, general ROS inhibition leads to decreased IL-1β (*left panel*) and IL-18 (*right panel*) secretion in simvastatin-treated cells when stimulated with LPS. The values are representative of four independent experiments. *B*, ratio of reduced *versus* oxidized glutathione in THP-1 cells. Simvastatin treatment leads to 50% reduction of ratio, as does the GGPP transferase inhibitor, indicating a more oxidized intracellular environment. The values are averages of five independent experiments. *C*, oxidative stress indicated by mRNA up-regulation of HO-1 (normalized to β2M). Simvastatin treatment increases oxidative stress and up-regulates HO-1. This can be partly rescued by co-incubation with the antioxidant *N*-acetylcysteine (*NAC*). The values are averages of three independent experiments. *D* and *E*, example of antioxidant capacity measurements of a single patient with its coupled healthy control. Gating on monocytes (*D*) and lymphocytes (*E*) was done using size and granularity. On the background measurements, the slopes are similar, indicating equal ROS production. *F*, simvastatin treatment leads to reduced antioxidant capacity in THP-1 cells. Shown is the ratio of the fluorescence increase with peroxide treatment divided by the background increase. A higher ratio is increased fluorescence and thus a lower capacity to prevent the probe from becoming oxidized. Each *line* represents an independent experiment. *G*, antioxidant capacity of blood monocytes from healthy controls (*HC*) *versus* MKD patients. The monocytes from patients have less capacity to clear oxidative stress. *H*, the difference in antioxidant capacity is specific for monocytes as MKD lymphocytes have a similar antioxidant capacity as healthy controls.

To verify the involvement of ROS in our model, we stimulated simvastatin-treated THP-1 cells with LPS for 4 h in the presence of three different indiscriminate ROS inhibitors (1 h preincubation): Apocynin, diphylene iodonium (DPI), and *N*-acetylcysteine. Generalized ROS inhibition normalized IL-1β and IL-18 secretion (Fig. 1*A*). To next assess the involvement of altered cytosolic redox status in our model, we compared glutathione concentrations in reduced and oxidized form (SH/SS ratio), in simvastatin-treated and control THP-1 monocytic cells. Simvastatin treatment lowers the SH/SS ratio by ∼50%, reflecting a shift in redox balance toward a more oxidative state. Inclusion of the geranylgeranylpyrophosphate (GGPP) transferase inhibitor recapitulates this reduction, suggesting that the decrease in SH/SS ratio is caused by a lack of non-sterol isoprenoids (Fig. 1*B*). Finally, oxidative stress causes the up-regulation of HO-1 mRNA levels (23). Indeed, simvastatin-treated cells express more HO-1 mRNA. This up-regulation can be partly rescued by the addition of the generalized ROS scavenger *N*-acetylcysteine (Fig. 1*C*). To investigate whether the increased oxidative burden is caused by increased ROS production or, alternatively, by decreased antioxidant capacity, we designed a new assay. In this assay we tested whether a lack of non-sterol isoprenoids impedes the ability to counter acute oxidative stress, in simvastatin-treated and nontreated cells. We added the general ROS-sensitive fluorescent probe H_2_DCFDA to cells and exposed them to increasing concentrations of H_2_O_2_. Cells that have diminished capacity to clear oxidative stress exhibit a sharper increase in fluorescence in response to H_2_O_2_. The background measurements (which is essentially ROS production) show donor variation, but there is no indication that simvastatin-treated cells have increased ROS production. The average slope of each sample is calculated and divided by the slope of the background (0 μm peroxide). This normalizes for the difference in ROS production between individuals (Fig. 1, *D* and *E*). Despite increased expression of HO-1, simvastatin-treated cells have impaired ability to clear acute oxidative stress, indicating diminished antioxidant capacity (Fig. 1*F*). Together, these data suggest that impaired non-sterol isoprenoid output is associated with a more oxidative cytosolic milieu because of diminishing antioxidant capacity and not because of increased ROS production.

To confirm these findings in actual patient cells, we isolated PBMC from seven MKD patients that did not experience fever at the time of sampling, each paired with a healthy control, as well as two additional healthy controls. We stained fresh PBMC with the H_2_DCFDA probe and gated on either monocytes or lymphocytes based on cell size and granularity features, using flow cytometry. MKD patient monocytes have a significantly reduced antioxidant capacity compared with monocytes from healthy control individuals (Fig. 1*G*). In contrast, lymphocytes from MKD patients and controls show comparable antioxidant responses (Fig. 1*H*). We conclude that isoprenoid-deficient monocytic cells have reduced antioxidant capacity.

##### Isoprenoid Deficiency Raises Mitochondrial Membrane Potential

**FIGURE 2.**
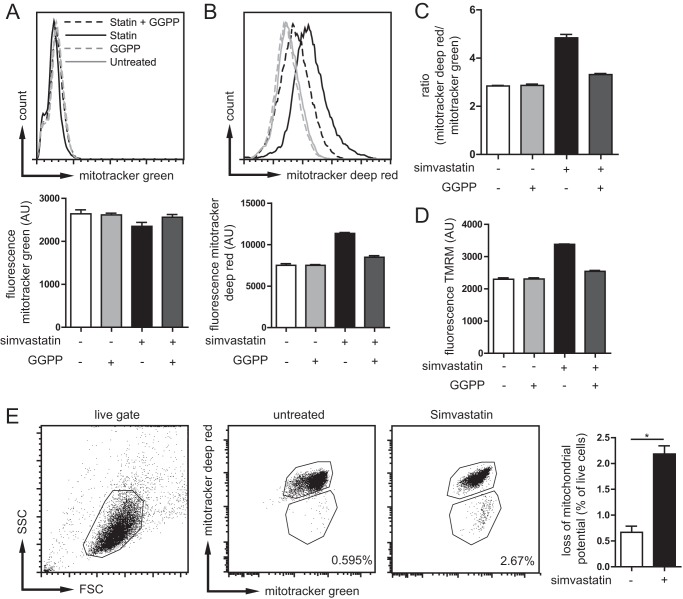
**Mitochondrial potential is altered in isoprenoid-deficient cells.** *A*, *top panel*, total mitochondrial mass as determined with MitoTracker Green in THP-1 cells under different conditions. *Bottom panel*, bar graph representation of data. Gating was done as shown in *E*, only top gate was used for potential measurements. *B*, *top panel*, mitochondrial potential determined by MitoTracker Deep Red staining. The potential is clearly increased in simvastatin treatment. *Bottom panel*, bar graph representation of data. *C*, ratio of mitochondrial potential over mitochondrial mass to more accurately determine the potential differences per unit of mitochondrial mass. Statin treatment leads to increased mitochondrial potential, which can be partially rescued by the addition of exogenous GGPP. *A–C* show the data of a representative experiment of five independent experiments, each consisting of triplicate measurements. *D*, staining of THP-1 cells with TMRM confirms MitoTracker data, the increased potential of mitochondria with simvastatin treatment, and dependence on isoprenoid availability. The data are representative of three independent experiments shown. *E*, increasing amounts of cells lose mitochondrial potential with simvastatin treatment as determined combined MitoTracker Green and Deep Red staining. The amount of cells (gated on live cells) losing mitochondrial potential is tripled by statin treatment. Representative plots are shown of four independent experiments. The *bar graph* shows the averages and S.E. of four independent experiments.

Our data thus far suggest that isoprenoid deficiency causes prolonged exposure to oxidative stress. We hypothesized that mitochondria, a source of ROS, might have reduced integrity in MKD, allowing leakage of ROS from the mitochondria. To address this possibility, we stained THP-1 cells with a fluorescent mitochondrial probe that reports the relative amount of mitochondria (MitoTracker Green) and, as a measure of mitochondrial damage, a mitochondrial probe that is sensitive to the mitochondrial inner transmembrane potential (MitoTracker Deep Red) (24). Damaged mitochondria lose membrane potential and lose MitoTracker Deep Red staining. Treatment with simvastatin resulted in an increase in transmembrane potential, as shown by increase in MitoTracker Deep Red staining, whereas the MitoTracker Green fluorescence was unchanged (Fig. 2, *A–C*, and gating strategy in *E*). Thus, the lack of isoprenoids leads to increased mitochondrial potential. Earlier studies had shown that an increase in mitochondrial transmembrane potential is associated with increased ROS production (25, 26). However, this work was done on isolated mitochondria. Other reports suggest that the regulation of mitochondrial potential can also be independent of ROS (27). To confirm the increase in mitochondrial potential in isoprenoid-deficient monocytes, we used a different probe, TMRM. Measurements with TMRM supported our MitoTracker results: simvastatin treatment increases mitochondrial potential. This is partially rescued by inclusion of GGPP, and therefore, an increase in mitochondrial potential is mediated by the lack of non-sterol isoprenoids (Fig. 2*D*). Although on average MitoTracker Deep Red fluorescence increased, the number of cells with intact MitoTracker Green fluorescence that lost MitoTracker Deep Red fluorescence altogether increased 3-fold, from 0.7% in untreated cells to 2.2% in simvastatin-treated THP-1 cells (Fig. 2*E*). This implies that the percentage of living cells that harbored damaged mitochondria increased 3-fold, as. Together, these data suggest that inhibition of protein isoprenylation is associated with increased mitochondrial transmembrane potential and a decrease in mitochondrial stability.

##### Damaged Mitochondria Accumulate in the Cytosol of Isoprenoid-deficient Monocytes

**FIGURE 3.**
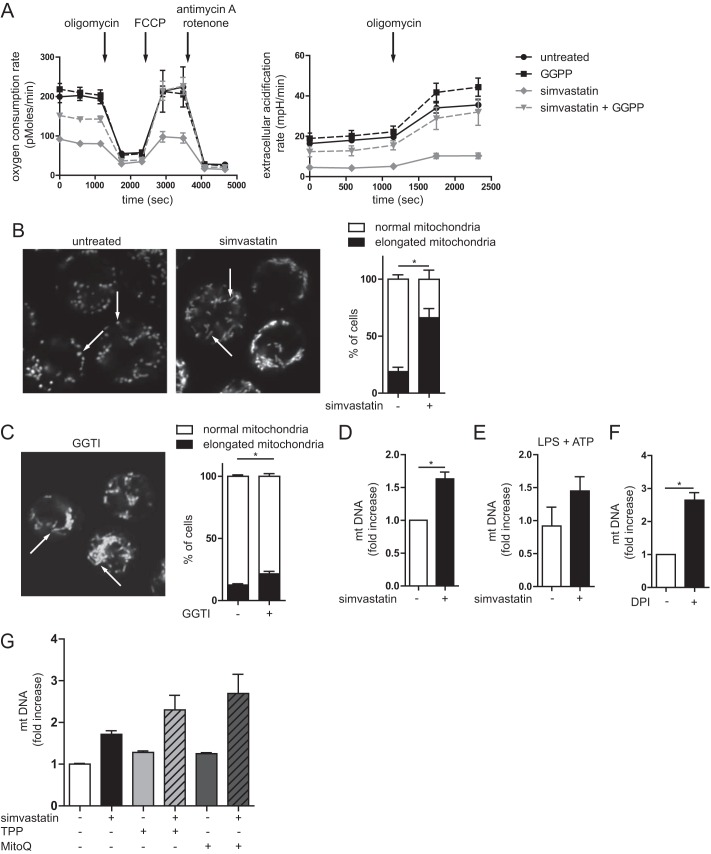
**Altered metabolism and release of mitochondrial components in isoprenoid-deficient cells.** *A*, oxygen consumption and glycolysis. Simvastatin-treated THP-1 cells have reduced metabolism, which is partly rescued by GGPP. Representative picture shown of three independent experiments. *B*, THP-1 cells are stained with MitoTracker Green and Deep Red and checked for mitochondrial morphology with confocal microscopy. Mitochondria in simvastatin-treated cells have a more elongated morphology. Representative pictures are shown. The bar graph consists of over 250 cells scored per condition; the *error bars* indicate variance in scoring by three different observers. *C*, to confirm that mitochondrial elongation is mediated by lack of isoprenylation, THP-1 cells were treated with GGPP transferase inhibitor (*GGTI*) (10 μm). This also leads to significant mitochondrial elongation, although not as strong as with simvastatin. The *bar graph* consists of over 150 cells scored per condition; the *error bars* indicate variance in scoring by three different observers. *D*, amount of mitochondrial DNA in the cytosolic fraction of THP-1 cells. Simvastatin treatment leads to an accumulation of mtDNA in the cytosol. The data are from four independent experiments combined. *E*, the accumulation of mitochondrial DNA is independent of cell activation. The data are the averages of two experiments shown. *F*, simvastatin does not induce the amount of cytosolic mtDNA as seen with DPI. The data are from four independent experiments combined. *G*, the release of mtDNA is not caused by ROS generated in the mitochondria. Co-incubation with mitochondrial ROS scavenger MitoQ cannot prevent the accumulation of mtDNA in the cytosol. TPP is used as control for mitochondrial targeting group in MitoQ. The averages of two independent experiments are shown.

We next investigated whether the increase in mitochondrial transmembrane potential is due to an increase in mitochondrial energy metabolism. To this end, we measured oxygen consumption and glycolysis rate. First basal respiration was measured, followed by addition of the ATPase inhibitor oligomycin to block respiration. Next, the uncoupler FCCP was added to induce maximum respiration, followed by the electron transport chain inhibitors antimycin A and rotenone (complex III and complex I, respectively) to completely abolish mitochondrial respiration. Simvastatin treatment caused a lower basal energy metabolism than that found in untreated cells, when assayed for either oxygen consumption or glycolysis rate (Fig. 3*A*, *left* and *right panels*, respectively). However, the regulation of oxygen consumption was similar under all conditions.

To assay for additional changes in mitochondria that may associate with the high transmembrane potential, we next visualized mitochondria in MitoTracker-stained THP-1 cells using confocal microscopy. The majority of simvastatin-treated cells exhibited mitochondria that were elongated, whereas in untreated cells, mitochondria appeared predominantly as small dots (Fig. 3*B*). To confirm that the elongation is due to prenylation and not to target effects of simvastatin, we repeated the experiment with the geranylgeranyl transferase inhibitor. This resulted in mitochondrial elongation, although not to the same extent. Earlier work showed that elongated mitochondria are associated with induction of autophagy (28) and that elongation of mitochondria modulates metabolic efficiency (29). Furthermore, isoprenoid deficiency caused by simvastatin treatment causes cells to proliferate more slowly. Taken together, isoprenoid deficiency in THP-1 cells causes both an increase in mitochondrial potential and mitochondrial elongation. It is possible that the mitochondrial elongation is responsible for the increase in membrane potential; however, this would need further investigation.

Mitochondrial components play an important role in priming immune responses (6, 7, 14). Because we observed mitochondrial irregularity and an increased proportion of cells harboring depolarized mitochondria, we hypothesized that isoprenoid-deficient monocytes contain released internal mitochondrial constituents. We therefore investigated the presence of mtDNA into the cytosol, by isolation of cytosolic fractions of cells and measured in these fractions the content of mtDNA (by quantitative PCR, assay for cytochrome *b* for which the coding sequence is located exclusively on mtDNA). We consistently found higher levels of mtDNA in cytosolic fractions in simvastatin-treated cells, both before (Fig. 3*D*) and after LPS/ATP stimulation (Fig. 3*E*), although the latter was not significantly different. The effect of simvastatin was pronounced, inducing nearly half the amount of mtDNA release seen in cells treated with the potent mitochondrial toxin DPI, which was used as a positive control (Fig. 3*F*). Raised mtDNA could be either actively released by the mitochondria or alternatively could be a consequence of defective autophagy-mediated clearance of damaged mitochondria. In mice, mtROS are necessary for the active release of mtDNA (11). To test whether the mtDNA is actively secreted by the mitochondria in human cells, we took a similar approach as Nakahira *et al.* (11) and co-incubated untreated and simvastatin-treated cells with the mitochondria-targeted antioxidant MitoQ, which is a ubiquinone targeted to the mitochondria by the triphenylphosphonium lipophillic cation. The non-antioxidant mitochondria targeting moiety TPP was used as a control (30). Both compounds show similar and even increased release of mtDNA into the cytosol (Fig. 3*G*), indicating that mtROS is not necessary to cause the mtDNA release in our culture system.

##### Accumulation of Defective Mitochondria Is Due to Impaired Autophagy

**FIGURE 4.**
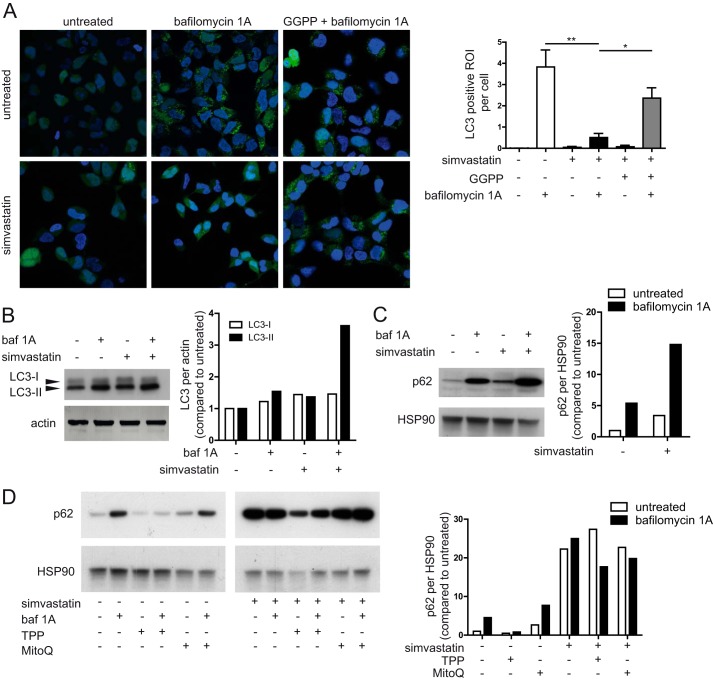
**Isoprenoid shortage causes deficiency in autophagy.** *A*, in HEK293 LC3-GFP cells, the lack of isoprenoids causes a clear reduction in the number of autophagosomes. This is almost completely dependent GGPP because the addition of exogenous GGPP can rescue the phenotype. Representative pictures are shown; the *bar graph* shows the averages of three experiments. *B*, in THP-1 cells, the defect appears not to be in the induction of autophagy because no differences are observed in LC3 levels. The *bar graph* contains quantification of shown blot, normalized first to loading control, and then the untreated condition is set to 1; the values are representative of four independent experiments. *C*, there is a late stage block in autophagy, as seen by the lack of p62 degradation upon simvastatin treatment. The *bar graph* contains quantification of shown blot, treated as in *B*, representative of four independent experiments shown. *D*, the defect in autophagy is not mediated by mtROS. The p62 levels after treatment with scavenger MitoQ or control compound TPP could not rescue the phenotype of the statin treatment. Protein levels in TPP conditions are lower because the concentration of TPP used approaches toxic levels. The *bar graphs* contains quantification of shown blot, normalized as in *B*, representative of two independent experiments shown.

Can defects in autophagy of mitochondria predispose human monocytes to IL-1β hypersecretion? Autophagy is controlled by multiple pathways, including those that are mediated by small GTPases. Because small GTPases are isoprenylated proteins, defects in the mevalonate pathway can lead to unprenylated GTPases (18, 31). We proposed that defects in isoprenylation may cause impairment of autophagy. We tested whether isoprenylation is required for generation of LC3^+^-mature autophagosomal membranes, using LC3-GFP fusion proteins expressed in HEK 293T cells (32). LC3 is a marker for autophagosomal membranes. Initially, it is an 18-kDa protein (LC3-I), which is matured to a 16-kDa form (LC3-II) when it is incorporated in the membranes. LC3-GFP cells were treated with simvastatin and/or the late stage autophagy inhibitor bafilomycin A1 (Baf A1). Baf A1 single treatment causes an accumulation of autophagosomes, whereas addition of simvastatin treatment to Baf A1 counteracts Baf A1-mediated autophagosome buildup (Fig. 4*A*). Thus, simvastatin treatment inhibits autophagosome formation as measured by LC3 autophagosomal membrane incorporation. This defect is caused specifically by defective isoprenylation because exogenously added GGPP rescues the phenotype.

To further analyze a possible role for autophagy in IL-1β hypersecretion by isoprenoid-deficient monocytes, we treated THP-1 cells with simvastatin (48 h) and prepared whole cell lysates for LC3 and p62 protein analysis by Western blot. LC3-II levels represent the induction of autophagy, whereas p62 degradation is a marker for successful completion of autophagy. Simvastatin, Baf A1 and their combined treatment led to increased levels of LC3-II in THP-1 cells (Fig. 4*B*), confirming earlier data that statins can induce autophagy (33, 34) and suggesting that the induction of autophagy is not inhibited by simvastatin. Levels of p62 were induced by Baf A1 treatment, as expected, because Baf A1 inhibits autophagosomal turnover of p62 and p62-associated protein aggregates (Fig. 4*C*). Statin treatment also caused an increase in p62, suggesting that statin inhibits autophagy at a stage between LC3-I/LC3-II conversion and successful completion of autophagy. Combined Baf A1 and simvastatin treatment resulted in a further increase in p62. To confirm that the increase in both p62 and LC3 is also isoprenoid-dependent, we repeated these experiments in the presence of exogenously added GGPP, to rescue its deficiency caused by simvastatin treatment. We conclude that inhibition of protein isoprenylation causes defective autophagosomal degradation in THP-1 cells.

We demonstrated that mtDNA release is not mediated by mtROS but wished to confirm that the mtROS released by defective mitochondria do not inhibit autophagy, supporting our hypothesis that lack of prenylation is the key factor in inhibited autophagy. Therefore, we specifically inhibited mtROS with the mitochondria-targeted antioxidant MitoQ, with TPP used as a control compound. We measured the protein levels of LC3 and p62 in monocytic cells. Again, MitoQ and TPP treatment did not change LC3 and p62 levels, negating a role for mtROS in the control of autophagy, with or without simvastatin pretreatment (Fig. 4*D*). This was confirmed in HEK293T LC3-GFP cells where the addition of MitoQ had no effect autophagosme formation (not shown). Antioxidant capacity of the MitoQ reagent itself was intact.^4^ We conclude the accumulation of damaged mitochondria originates from a defect in autophagy and leads to the release of mitochondrial components. Inhibition of mtROS cannot prevent the damage to mitochondria nor rescue the autophagy defect in these cells.

##### IL-1β Secretion Involves Both Cytosolic Release of Mitochondrial Components and Defective Autophagy

**FIGURE 5.**
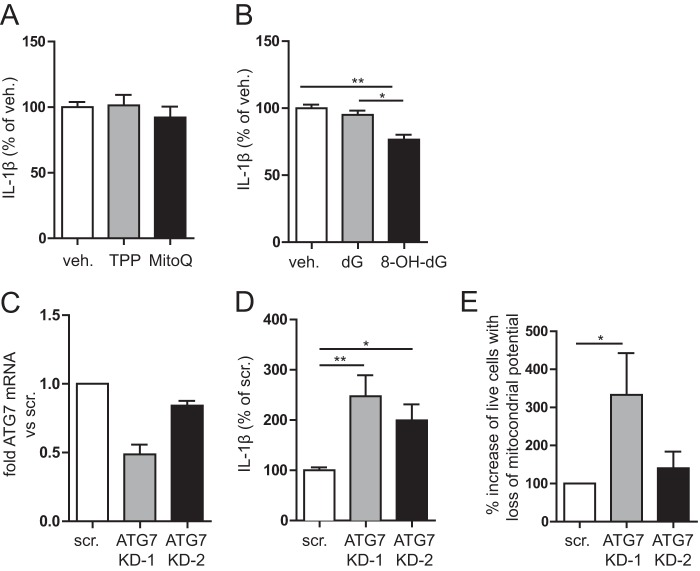
**IL-1β secretion in isoprenoid-deficient cells involves inflammasome activation by oxidized mitochondrial DNA and defective autophagy.** *A*, IL-1β secretion in MKD model is not mediated directly by mtROS. Despite MitoQ pretreatment, it did not ameliorate IL-1β secretion. *B*, inflammasome activation is mediated by oxidized mtDNA. Incubation of simvastatin-treated cells with oxidized nucleotides prior to stimulation leads to decreased IL-1β secretion, whereas non-oxidized nucleotide has no effect. The averages of three independent experiments are shown. *C*, knockdown of ATG7 by two virally stably transduced shRNAs in THP-1 cells. The amount of mRNA for ATG7 was normalized to GAPDH and compared with scrambled control cells. *D*, IL-1β secretion of ATG7 KD cells after stimulation with LPS and ATP. IL-1β secretion is clearly increased in cells with impaired autophagy. This was done without simvastatin pretreatment to isolate the effect of defective autophagy. The averages of three experiments are shown. *E*, defective autophagy leads to accumulation of damaged mitochondria. ATG7 KD cells were either treated with DPI or left untreated, followed by staining with MitoTracker Green and Deep Red. Cells were gated as done for Fig. 2*E*. When autophagy is defective, induction of mitochondrial stress leads to accumulation of damaged mitochondria. The *bar graph* with averages of four independent experiments is shown. *veh.*, vehicle; *scr.*, scrambled.

Is the release of mitochondrial components into the cytosol necessary for IL-1β release? The selective inhibition of mtROS using MitoQ counteracts proinflammatory cytokine secretion, in the autoinflammatory disease TRAPS (7). We used MitoQ in simvastatin-treated THP-1 monocytes to determine whether mtROS are required for LPS-induced IL-1β secretion. In cells treated with MitoQ, the IL-1β secretion upon LPS stimulation was unaffected (Fig. 5*A*). We conclude that ROS, but not mitochondrial ROS, are involved in the IL-1β secretion in isoprenoid deficiency or MKD.

Oxidized mtDNA serves as activating ligand for the NLRP3 inflammasome (14). Because we observed that isoprenoid depletion significantly increased mtDNA levels in the cytosol (Fig. 3*C*), we hypothesized that the mtDNA is oxidized and activates the NLRP3 inflammasome, thereby increasing IL-1β secretion. The binding of oxidized mtDNA to NLRP3 and subsequent inflammasome activation can be competitively inhibited by oxidized nucleotides that do not induce inflammasome activation (14). We incubated simvastatin-treated THP-1 cells for 1 h with either oxidized deoxyguanosine or regular deoxyguanosine, followed by 4 h of LPS stimulation. We found that oxidized deoxyguanosine significantly reduced the amount of secreted IL-1β compared with both vehicle and deoxyguanosine control (Fig. 5*B*). Thus, mtDNA release contributes to the IL-1β hypersecretion via the NLRP3 inflammasome. Having shown that isoprenoid shortage causes both IL-1β generation *and* impaired autophagy, we hypothesized that isoprenoid shortage causes IL-1β generation *through* impaired autophagy. We therefore transduced THP-1 monocytic cells with shRNA KD constructs for the autophagy protein ATG7 or a scrambled control. We generated two different THP-1 lines with 50 and 20% KD efficiency (Fig. 5*C*). Cells were stimulated with LPS and ATP, and the level of secreted IL-1β was measured (Fig. 5*D*). As expected, both KD lines secreted more IL-1β than the scrambled control, with the 50% KD secreting more than the 20% KD. This confirmed that impairment of autophagy enhances IL-β secretion, irrespective of isoprenoid biosynthesis. To finally confirm that the defective autophagy is responsible for the accumulation of damaged mitochondria, we induced mitochondrial damage in the ATG7 KD and control THP-1 cells as described (4-h DPI (15 μm) treatment (35)) and measured the fraction of cells that lose mitochondrial potential in ATG7 KD and control cells. Loss of potential was gauged by flow cytometry using cell staining with MitoTracker Green and MitoTracker Deep Red. Using the same gating strategy as for Fig. 2*E*, we observed that ATG7 KD samples exhibit an increase in live cells that are losing mitochondrial potential (Fig. 5*E*). The increase is evident in the 50% ATG7 KD cells yet did not reach significance in the 20% ATG7 KD cells. Thus, defective autophagy results in the accumulation of damaged mitochondria in THP-1 cells.

##### Defective Autophagy in MKD Monocytes Leads to Increased IL-1β Secretion

**FIGURE 6.**
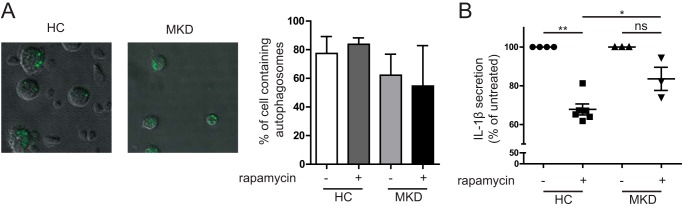
**Primary MKD cells respond differently to autophagy stimulus.** *A*, freshly isolated PBMCs from a healthy donor and a MKD patient were stained with an autophagy probe. Samples were checked for number of cells with autophagosomes and autophagosomes per cell, but no significant differences were observed, largely because of significant variation in healthy controls (*HC*). Representative pictures show autophagosomes in both HC and MKD patients. The *bar graph* shows quantification of the percentage of monocytes containing one or more autophagosomes (healthy controls, *n* = 3; patients, *n* = 2). *B*, PBMCs from healthy donors and MKD patients were stimulated for 1 h with rapamycin (250 nm) followed by 4 h of LPS (200 ng/ml) stimulation. Inducing autophagy by rapamycin leads to a decrease of IL-1β secretion in healthy controls of approximately 40%, whereas in MKD patients the reduction is less than 20%. This indicates that inducing autophagy has less of an effect in IL-1β secretion in MKD patients. *, *p* < 0.05; **, *p* < 0.01.

To confirm our findings of defective autophagy in isoprenoid-deficient THP-1 cells, we investigated autophagy in fresh MKD patient-derived monocytes, by visualization of autophagosomes and testing their ability to modulate levels of IL-1β secretion. We stained autophagosomes in PBMCs from both healthy controls and MKD patients. MKD patient monocytes display a partial block in autophagy, confirming our work in simvastatin-treated THP-1 cells. Autophagosomes are visible in both types of monocytes (Fig. 6*A*). Treatment of these monocytes with the autophagy-inducing agent rapamycin does not modify the number of cells with autophagosomes or autophagosomes per cell. However, there is a clear difference in the IL-1β secretion between healthy controls and patient cells treated with rapamycin. In healthy controls, preincubation of PBMCs with rapamycin (250 nm) 1 h prior to LPS stimulation (4 h) significantly reduces IL-1β secretion (*p* < 0.01, *n* = 5). In contrast, the reduction of IL-1β secretion in rapamycin-treated MKD PBMCs did not reach significance (*n* = 3; Fig. 6*B*, *ns*). This indicates that inducing autophagy counteracts IL-1β cytokine secretion in healthy controls. This is in line with previous studies done in mice, where rapamycin-treated mice had lower IL-1β serum levels after LPS challenge (36). In MKD patients, where autophagy is already defective, inducing autophagy has limited effect. Taken together, our data suggest that inflammasome activation in MKD, can involve damaged mitochondria in monocytes. Because of defects in autophagy, these damaged mitochondria are not effectively cleared, resulting in accumulation of mitochondrial components in the cytosol. These components, left present in the cytosol when autophagy is defective, prime monocytes in MKD for Il-1β hypersecretion.

## DISCUSSION

We here addressed a role for autophagy in the development of autoinflammation in the periodic fever syndrome, MKD. Autophagy is a cellular process for the turnover of damaged proteins and organelles, including mitochondria (12). It had been known that both insufficient and excessive autophagosomal degradation is harmful for the cell. Autophagy is therefore strictly regulated by several signaling pathways that are inhibitory (serine-threonine kinase mammalian target of rapamycin and class I PI3Ks) or that activate autophagy (class III PI3Ks) (38). Isoprenylation can modulate the induction of autophagy as was suggested from experiments in which statin treatment-induced autophagy (33, 39, 40). However, dissimilarities in statin dosage and treatment protocols complicate the interpretation of these studies. Also, the induction was not seen in all models, suggesting that statin-induced autophagy is cell type-specific. Our results here show a direct role for isoprenylation in the induction and completion of autophagy in monocytic cells. We show that autophagy controls IL-1β release through removal of mitochondrial components that otherwise stimulate NLRP3 inflammasome activation. A strong genetic link was recently described between the autophagy gene ATG16L1 and susceptibility to chronic inflammation in Crohn disease (41, 42). ATG16L1 suppresses IL-1β signaling by promoting the degradation of autophagosomal p62 (43). In addition, a recent study by Bachetti *et al.* (21) showed that in TRAPS, a mutant version of TNF receptor 1 causes inhibition of autophagy, leading to autoinflammatory disease. In this current study, we provide experimental proof that general autophagic dysfunction enhances IL-1β release.

Although the effect of isoprenoid deficiency on autophagy is clear, there are still some unresolved issues. Despite our best efforts, we have not been able to explain the increased mitochondrial potential associated with isoprenoid deficiency. This effect is highly reproducible yet seems contradictory with the low oxygen metabolism. It is possible that the elongation of the mitochondria, associated with increased efficiency, leads to a higher potential even with lower oxygen consumption rates. To clarify this issue, mitochondrial elongation should be induced without the starvation effect of simvastatin. Unfortunately, we are unable to do so in our model system.

Recent work shows a role for ROS in the development of the IL-1β-driven autoinflammatory disorders (7, 16, 44). Initially, NADPH-oxidase-derived ROS production was also implicated in IL-1β release in the periodic fever cryopyrin-associated periodic syndrome, but analyses of chronic granulomatous disease patients discounted a role for NADPH-oxidases in these disorders (16, 17). The source of ROS appeared mitochondrial (*i.e.*, mtROS) rather than derived from the NADPH-oxidases at the plasma membrane, as was supported by work in cells from chronic granulomatous disease patients that have defective NADPH-dependent ROS caused by mutations in p47*^phox^* (16). A publication on TRAPS showed that specific inhibition of mtROS release alleviates cytokine secretion (6). Our own data here show that monocytes of MKD patients exhibit defective antioxidant responses, whereas MKD lymphocytes responded similarly to healthy control cells when exposed to acute redox stress. Although we can inhibit cytokine secretion using general ROS inhibitors, the specific inhibition of mtROS had no effect on the cytokine secretion. The question that remains is what is the source of ROS that mediates the hypersecretion? It is possible that the hypersecretion of IL-1β is mediated by mtROS and that the (membrane-bound) MitoQ is unable to inhibit all ROS escaping from compromised mitochondria. However, additional research will be needed to confirm or discount this possibility. The notion that ROS play a role in statin-induced IL-1β generation has been recently supported by similar findings in simvastatin- and fluvastatin-treated human monocytes and murine macrophages (24).

Under conditions of oxidative stress, thioredoxin-interacting protein (TXNIP) associates to NLRP3, as was shown in human THP-1 cells. The presence of ROS in the cytosol was thereby directly linked to inflammasome activation and secretion of bioactive IL-1β (9). Our work supports the earlier reported requirement for cytosolic ROS in IL-1β release by monocytes (7, 9, 44) and adds that mitochondrial alterations and defects in autophagy increase mtDNA into the cytosol. Our data thereby place autophagy upstream of mtROS release and NLRP3 inflammasome activation. The defective autophagy cannot clear damaged mitochondria, which leads to release of mtROS and mtDNA in the cytosol, which in turn can activate the NLRP3 inflammasome. We observed that increased IL-1β release is associated with reduced mitochondrial activity (*i.e.*, attenuated respiratory chain activity) and mtROS and mtDNA cytosolic release. The cytosolic presence of oxidized mtDNA release contributes directly to IL-1β generation, which supports earlier work showing that cytosolic mtDNA directly stimulates NLRP3 inflammasomes in mouse macrophages (11). Recent work shows the formation of autophagosomes at contact sites between mitochondria and endoplasmic reticulum, further corroborating a role for autophagosomes in clearance of mitochondrial constituents (37). Mouse macrophages that are deficient in autophagy proteins beclin-1 and LC3B secrete increased levels of IL-1β upon NLRP3 inflammasome activation using LPS/ATP combined treatment (11). Mouse macrophages that lack mtDNA because of culture in the presence ethidium bromide exhibit reduced caspase-1 activity and attenuation of respiratory chain activity and ROS production. The generation of mtROS was placed as an upstream mechanism in the release of IL-1β. However, NLRP3 inflammasome stimuli may differ in their subcellular source of ROS (*i.e.*, crystalline particles such as asbestos and silica may induce NLRP3 inflammasome activation through ROS production by NADPH-oxidase (9, 45)). Accordingly, mito-TEMPO, which inhibits mtROS release, did not inhibit IL-1β secretion induced by LPS and monosodium urate (11). Our own findings in human cells support these data, because our block of mtROS release using MitoQ did not inhibit IL-1β release.

Defective autophagy had earlier been linked to IL-1β release; depletion of autophagy proteins promotes NLRP3-mediated IL-1β release via the release of mtDNA and ROS in mouse macrophages (11). Moreover, *Mycobacterium tuberculosis*-infected ATG7 KD macrophages secrete more IL-1β (46). In periodic fever disorders, a role for defective autophagy had not yet been established. We here show, for the first time, the complex interplay of autophagy and mitochondrial function and how imbalance of this interplay leads to excessive IL-1β generation, in a well established model of the autoinflammatory disease MKD. Furthermore, our data with fresh primary PBMC from MKD patients confirm the data found in the MKD model system. Together, our results support that defective autophagy plays a role in the pathogenesis of this autoinflammatory disease and possibly that of other periodic fever syndromes as well.

## Footnotes

4 R. van der Burgh, L. Nijhuis, K. Pervolaraki, E. B. Compeer, L. H. Jongeneel, M. van Gijn, P. J. Coffer, M. P. Murphy, P. G. Mastroberardino, J. Frenkel, and M. Boes, unpublished results.

## Footnotes

3 The abbreviations used are:

ROS: reactive oxygen species
MKD: mevalonate kinase deficiency
TRAPS: TNF receptor 1-associated periodic syndrome
mtDNA: mitochondrial DNA
TMRM: tetramethyl rhodamine methyl ester
TPP: decyltriphenylphosphonium
PBMC: peripheral blood mononuclear cell
KD: knockdown
mtROS: mitochondrial ROS
DPI: diphylene iodonium
GGPP: geranylgeranylpyrophosphate
HO-1: heme oxygenase-1
Baf A1: bafilomycin A1
H_2_DCFDA: 2′,7′-dichlorodihydrofluorescein diacetate.
